# No bones about it: regulatory T cells promote fracture healing

**DOI:** 10.1172/JCI188368

**Published:** 2025-01-16

**Authors:** Jason W. Griffith, Andrew D. Luster

**Affiliations:** 1 Center for Immunology and Inflammatory Diseases,; 2 Division of Pulmonary & Critical Care Medicine, and; 3 Division of Rheumatology, Allergy & Immunology, Massachusetts General Hospital, Harvard Medical School, Boston, Massachusetts, USA.

## Abstract

Regulatory T cells (Tregs) are increasingly being recognized for their role in promoting tissue repair. In this issue of the *JCI*, Chen et al. found that Tregs at the site of bone injury contribute to bone repair. The CCL1/CCR8 chemokine system promoted the accumulation of Tregs at the site of bone injury, where Tregs supported skeletal stem cell (SSC) accumulation and osteogenic differentiation. CCL1 increased the transcription factor basic leucine zipper ATF-like transcription factor (BATF) in CCR8^+^ Tregs, which induced the secretion of progranulin that promoted SSC osteogenic function and new bone formation. This study highlights the ever-expanding role of Tregs in tissue repair by demonstrating their ability to expand stem cells at a site of injury.

## Bone injury and innate and adaptive immunity

Bone fracture is a common event, with over 170 million fractures per year globally (1). An estimated 2% to 10% of these fractures go onto nonunion, which is defined by the FDA as a fracture that persists for more than nine months without signs of healing progress for three months, thus lacking the potential to heal without further intervention (2). Risk factors for nonunion are multifactorial and depend on the bone affected, but generally include the severity of the injury, impaired blood supply, age, body mass index, diabetes, osteoarthritis, autoimmune disease, HIV, cigarette smoking, excessive alcohol use, and medications such as opioids, nonsteroidal antiinflammatory drugs, antibiotics such as fluoroquinolones, and anticoagulants such as warfarin (3). Treatment for nonunion often requires surgical intervention with addition or replacement of internal fixation (4). Additional biologic stimulation with bone morphogenic protein (BMP) is approved for long bone nonunion, and systemic parathyroid hormone therapy (teriparatide) may also be used (2). Despite these therapies, nonunion is associated with reduced health status due to greater physical impairment and pain, results in increased unemployment rates as much as a year after the fracture, and more than doubles the cost of total care (5). Thus, new therapeutic approaches to promote fracture repair are still needed.

Fracture healing is a complex process, characterized by hematoma formation and an early inflammatory phase, a repair phase with cartilaginous callus and bony callus formation, and remodeling (6). In the last decade, the role of immune cells in orchestrating this complex process has been increasingly appreciated. Within minutes after a fracture occurs, platelets and innate immune cells, such as neutrophils and macrophages, populate the injury and produce proinflammatory cytokines and chemokines, while in later stages, neutrophils and macrophages remove cellular and tissue debris and acquire an antiinflammatory phenotype (6). Highlighting the roles of these innate cells, depletion studies of either macrophages or neutrophils in mouse models of fractures results in higher rates of nonunion (7, 8). In the later stages of the inflammatory phase, adaptive T cells arrive at the fracture site and regulate osteoblast-osteoclast equilibrium by secretion of cytokines, such as IFN-γ, TNF-α, and receptor activator of nuclear factor-κβ ligand (RANKL) (6). Regulatory T cells (Tregs), characterized by the expression of the transcription factor Foxp3, have also been observed at fracture sites, and decreased Tregs have been observed in patients with nonhealing fractures (9). Recently, punctual deletion of Tregs using the anti-CD25 antibody has been shown to compromise fracture healing with increased fracture gap and lower callus volume, but the mechanism by which Tregs promote healing has not been fully elucidated (10).

## CCL1/CCR8 signaling in Tregs and progranulin-mediated bone healing

In this issue of the *JCI*, Chen et al. (11) found that Treg numbers were increased by approximately three-fold at the site of bone injury. Treg numbers peaked seven days following drill-hole surgery of the femur and gradually returned to baseline after one month. Treg depletion using *Foxp3*-DTR mice revealed an important role for Tregs in bone healing with a reduction of deposited bone matrix and cartilage tissue in the injury lesion on day 14. Treg-depleted mice also showed persistent nonunion with primarily fibrous tissue around the injury site. These findings were verified in a femur fracture model in mice. Injury induced the expansion and activation of skeletal stem cells (SSCs) within the injury site, which gave rise to abundant skeletal progenitor cells and mature osteoblasts for bone repair. Treg-depleted mice had decreased percentages of total skeletal stem lineage cells, SSCs, stromal progenitor cells (BCSPs), and mature osteo-lineage cells within the callus. These results indicated that injury-induced SSC niche expansion was diminished under the Treg-depletion condition.

**Figure 1:**
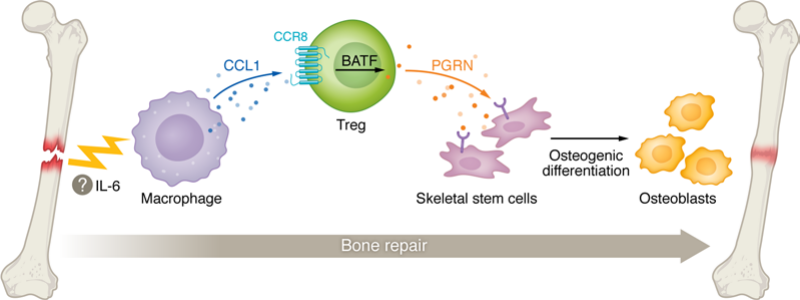
Tregs at the site of bone injury contribute to bone repair via the CCL1/CCR8 axis. Bone fracture induces CCL1 production from bone marrow macrophages, resulting in the accumulation of CCR8^+^ Tregs at the injury site. CCL1/CCR8 signaling in Tregs induces the expression of the transcription factor BATF, which in turn induces PGRN secretion. PGRN promotes skeletal stem cell differentiation, osteogenic function, and new bone formation.

Bulk RNA-Seq analysis of SSCs from the callus revealed that Treg-depleted mice downregulated gene expression related to stemness and osteogenesis while upregulating the expression of genes associated with apoptosis and senescence. Notably, bone injury induced a phenotypic change of Tregs at the injury site, including upregulation of the chemokine receptor CCR8, which has been associated with tissue Tregs in other settings. In an effort to determine how the CCR8^+^ Tregs accumulated at the injury, Chen and authors treated mice with FTY720, an S1P1 receptor antagonist that inhibits lymphocyte egress from tissue. Reductions in the percentage of CCR8^+^ Tregs at the injury site suggested that peripheral CCR8^+^ Tregs contributed to their accumulation. The chemokine ligand for CCR8, CCL1, was highly induced in macrophages within the wound/callus tissue and was detectable on day three, sustaining expression to day 14 after injury. Mice treated with a CCR8 inhibitor or a CCL1-mAb showed markedly reduced proportions and numbers of Tregs compared with untreated mice on day seven after injury and this reduction was associated with impaired bone repair. These findings suggest that the CCL1/CCR8 chemokine axis drove Treg recruitment into the injured tissue. CCL1 was mainly produced by lesional macrophages, perhaps in response to IL-6, and induced the expression of the basic leucine zipper ATF-like transcription factor (BATF) from CCR8^+^ Tregs. In the injured tissue, CCR8^+^ Tregs upregulated the osteogenic factor progranulin (PGRN), which was shown to be important for inducing SSC osteogenic differentiation in vitro, supporting bone repair (Figure 1) (11).

This interesting study adds to the growing literature that tissue Tregs regulate tissue homeostasis in response to injury and are an important source of growth and reparative factors that aid in the tissue-repair process (11). CCR8 is highly expressed on tissue Tregs; however, it does not appear to uniformly play a role in Treg recruitment into tissue. For example, while Tregs in tumors highly express CCR8, it does not appear to contribute to their recruitment into tumors nor does it appear to act in a suppressive capacity (12, 13). It is not clear why CCR8 plays a role in Treg recruitment and function in some tissue but not in others. It should be noted that Chen et al. did not formally demonstrate that CCR8 mediated Treg recruitment into the injured bone. CCR8^+^ Treg numbers increased at the injury site along with decreased numbers of CCR8^+^ Tregs in the adjacent lymph node. FTY720 treatment inhibited these changes, which suggests CCR8^+^ Tregs migrated from the periphery into the injury site. While this experiment is informative, it is not direct proof of Treg migration, as Tregs from the lymph node or peripheral blood were not directly traced into the injured bone. CCL1/CCR8 signaling in Tregs at the injury site may also contribute to their increased accumulation by inducing proliferation and/or cell survival as has been seen in group 2 innate lymphoid cells (14) with the net result being increased accumulation. If Tregs are recruited into the injury site, it will of interest to determine their origin. While Chen and authors speculate that the Tregs are recruited from the adjacent lymph node, recent studies suggest tissue Tregs can be recruited to sites of injury from other tissue sites, such as the gut (15).

## Tregs as protectors/promotors of stem cell populations

The findings by Chen et al. that Tregs promote bone fracture healing via PGRN-mediated osteogenic differentiation of SSCs adds to the growing literature that Tregs are key players in tissue repair in various organs via specialized interactions with tissue-resident stem cells. In the skin, Tregs preferentially localize to the hair follicle (HF) where they support HF regeneration via expression of Jagged 1 to promote HF stem cell (HFSC) function and activation (16). During epidermal skin injury, Tregs suppress IL-17A production and downstream CXCL5-mediated neutrophil recruitment, modulating excessive inflammation to allow for efficient HFSC differentiation and migration to the site of injury and thereby contributing to the restoration of skin-barrier integrity (17). During virally mediated lung injury, Tregs promote tissue repair by production of the EGFR ligand amphiregulin, which activates adventitial fibroblast production of growth factors promoting the differentiation of type II alveolar epithelial (AT2) cells into airway lining type I alveolar epithelial cells (17). Like those in the skin, Tregs in the lung also suppress neutrophil recruitment via suppression of CXCL5 production (in this case by adventitial fibroblasts themselves) and thus it is tempting to speculate that Tregs protect the adventitial fibroblast-AT2 niche from excessive inflammation, although this has not yet been definitively shown (18). In the small intestine, the ablation of Tregs reduced the Lgr5^+^ intestinal stem cell (ISC) pool and promoted the accumulation of differentiated epithelial cells, suggesting that Tregs maintain the ISC niche via production of IL-10 (19). Taken together, these studies indicate that Tregs are critical for tissue repair via modulation of inflammatory responses in the stem cell niche and via the production of direct factors, such as PGRN in the bone, Jagged-1 in the skin, and amphiregulin in the lung and muscle, that directly promote tissue stem cell function. Given that PGRN is protective in a variety of neurodegenerative diseases (20), it will be of interest to determine if PGRN expression by Tregs is unique to the bone or if this phenomenon occurs in other tissues, such as the brain.

## Clinical implications

Whether augmentation of Treg function can be used to promote bone healing will require more study. However, it should be noted that CCR8 antibody–mediated depletion of Tregs has been shown in many preclinical models to improve antitumor immunity and this strategy is actively being developed for human therapy (12, 13). The results of Chen et al. (11) suggest that CCR8^+^ tissue Tregs play an important role in protecting tissue stem cells and in tissue repair and these roles will need to be evaluated in clinical trials that evaluate these new CCR8 antibody therapies.

## Version 1 01/16/2025

Electronic publication
